# Fetal Cortical Layers and Midline Histological Alterations in the BTBR Mouse Model of Neurodevelopmental Disorders

**DOI:** 10.3390/biology15161425

**Published:** 2026-08-18

**Authors:** Joanna Czyrska, Piotr Poznański, Agnieszka Bernat, Dawid Winiarczyk, Marta Marlena Ziętek, Silvestre Sampino

**Affiliations:** 1 Department of Experimental Embryology, Institute of Genetics and Animal Biotechnology of the Polish Academy of Sciences, Jastrzębiec, 05-552 Magdalenka, Poland; j.czyrska@igbzpan.pl (J.C.); a.bernat@igbzpan.pl (A.B.); d.winiarczyk@igbzpan.pl (D.W.); m.zietek@igbzpan.pl (M.M.Z.); 2 Department of Biotechnology and Nutrigenomics, Institute of Genetics and Animal Biotechnology of the Polish Academy of Sciences, Jastrzębiec, 05-552 Magdalenka, Poland; p.poznanski@igbzpan.pl; 3 Laboratory of Photobiology and Molecular Diagnostics, Intercollegiate Faculty of Biotechnology, University of Gdansk and Medical University of Gdansk, 80-309 Gdansk, Poland

**Keywords:** autism spectrum disorder, prenatal brain development, corpus callosum, forebrain cortex, midline glia

## Abstract

The BTBR T+ Itpr3tf/J (BTBR) strain is a widely used model of neurodevelopmental disorders, characterized by an altered neuroanatomy, including a full-penetrant agenesis of the corpus callosum. While the adult BTBR brain has been studied thoroughly, less is known about how its brain develops prenatally and about when neurodevelopmental trajectories begin to differ from neurotypical strains. Here, we conducted a comparative histological analysis of fetal brain development across multiple developmental stages in BTBR and C57BL/6J (B6) mice, focusing on neocortical and midline development. BTBR mice showed lower fetal weight but comparable somite counts (assessed at 12.5 days post coitum, dpc) compared to B6 controls, indicating similar developmental timing. Neocortical layering showed a reduced ventricular zone fraction in BTBR fetuses at 15.5 dpc, without a change in overall cortical thickness. Concurrently, midline cell populations immunoreactive for the glutamate aspartate transporter (GLAST) failed to undergo remodeling in subsequent stages, resulting in the failure to form a GLAST+ indusium griseum and the lack of callosal projections crossing the midline at 17.5 dpc, which instead develop normally in B6 fetuses. These findings offer structural correlates for the early neuropathology of the BTBR brain.

## Simple Summary

Brain differences linked to autism are thought to begin before birth, but the earliest steps are difficult to observe. Using a mouse strain previously reported to show autism-like behavior and lacking the connection between the two halves of the brain, we examined developing brains at several stages alongside typically developing mice. Overall, developmental timing was unaffected, yet the reshaping of the brain’s midline that normally allows the two sides to connect did not take place. Pinpointing this early failure shows when such brain-wiring differences first arise.

## 1. Introduction

Autism spectrum disorder (ASD) is a heterogeneous neurodevelopmental condition characterized by social communication difficulties, restricted behaviors, and atypical sensory processing. Converging evidence suggests that deviations in brain development arise during prenatal life and set the stage for later emerging behavioral phenotypes. Identifying such early alterations is therefore essential for understanding ASD pathogenesis and for developing strategies aimed at early diagnostic or prophylactic intervention [1,2]. In humans, quantitative reduction of corpus callosum area is among the more consistently replicated structural findings in autism [3], whereas frank callosal agenesis is rare in unselected autism cohorts and is not a diagnostic criterion [4,5].

Mouse models play a central role in dissecting the etiology and pathogenesis of neurological and neuropsychiatric disorders. Among them, ASD, schizophrenia, attention deficit hyperactivity disorder (ADHD), and many congenital neurodevelopmental disorders are known to be linked with prenatal disruptions of neuronal and glial fetal structures and function [6,7]. The BTBR T+ Itpr3tf/J (BTBR) strain is widely used because it robustly expresses autism-like behaviors, including impaired sociability, repetitive grooming, and atypical ultrasonic vocalizations, and because it exhibits striking neuroanatomical anomalies, most notably a complete agenesis of the corpus callosum (CC), in comparison to other inbred strains, such as the C57BL/6J (B6) mouse [8,9,10,11,12,13]. Mouse models of autism differ substantially in their neuroanatomical phenotypes. Comparative magnetic resonance imaging of 26 such models found that they fall into three neuroanatomical clusters, two of which relate to altered connectivity patterns reported in autism, with BTBR falling in the same cluster as the *Nlgn3* and *Slc6A4* mutant lines [14]. Models carrying a single defined mutation, such as *Shank3*, *Fmr1* or *Cntnap2* mice, address the consequences of one identified genetic lesion. In comparison, in the present study, BTBR was chosen because of its polygenic nature, in which agenesis of the corpus callosum has been reported as fully penetrant [15]. Previous studies on BTBR mice have focused on the postnatal or adult brain, documenting callosal agenesis, abnormal cortical connectivity, and behavioral phenotypes [16]. In these mice, the CC is completely absent, and the hippocampal commissure is frequently reduced or missing, resulting in pronounced deficits in interhemispheric connectivity [10]. Previous studies suggest that these commissural defects are accompanied by alterations in the interhemispheric fissure (IHF), the midline structure that normally guides callosal formation [17,18,19,20]. Failure of IHF remodeling is a recognized mechanism of callosal agenesis across eutherian mammals, including humans [21]. Midline astroglia and extracellular matrix remodeling are known to be critical for IHF remodeling and CC formation [21,22]. In this study, we examined prenatal cortical and midline development in BTBR and B6 mice to identify early structural features associated with the BTBR callosal phenotype. We compared neocortical compartment proportions during mid-gestation, assessed IHF remodeling, and analyzed the organization of midline cell populations immunoreactive for the glutamate aspartate transporter (GLAST) across embryonic stages. Our analyses reveal strain-specific differences in cortical compartmentalization, midline remodeling, and GLAST+ cell organization that emerge during fetal development. Together, these findings highlight altered prenatal cortical development and midline GLAST+ cell organization as potential candidate contributors to the neuroanatomical phenotype in the BTBR strain.

## 2. Materials and Methods

### 2.1. Experimental Design

Multiple developmental outcomes were analyzed in BTBR and B6 fetuses at different stages: (i) fetal body weight at 12.5, 15.5, and 17.5 days post coitum (dpc) and somite counts at 12.5 dpc; (ii) total cortical thickness at 12.5 and 15.5 dpc; (iii) relative thickness of the cortical compartments: ventricular zone (VZ), intermediate zone (IZ), and cortical plate (CP) at 15.5 dpc [23]; (iv) IHF length at 15.5 and 17.5 dpc; (v) immunohistochemical analysis of GLAST-immunoreactive (GLAST+) cell populations at 15.5 and 17.5 dpc; and (vi) adult CC. Developmental stages were selected according to the specific outcome measured. At 12.5 dpc, somite number is still externally resolvable and can therefore be used as a staging index, and the neocortex is at the onset of laminar organization. At 15.5 dpc, the ventricular zone, intermediate zone and cortical plate can each be distinguished on the basis of nuclear density, and this stage was therefore used for the analysis of cortical compartment proportions. In the mouse, remodeling of the interhemispheric fissure begins at approximately embryonic day 15 and is largely complete by day 18 of gestation [22]. Measurements of interhemispheric fissure length and the immunohistochemical analysis of GLAST-immunoreactive midline cells were therefore performed at 15.5 and 17.5 dpc.

### 2.2. Animals and Housing

The experimental animals used in this study belonged to the autism-like BTBR T+ Itpr3tf/J (BTBR) strain and the standard neurotypical strain C57BL/6J (B6). Mice were originally obtained from The Jackson Laboratory (Bar Harbor, ME, USA) and subsequently maintained in the animal facility of the Institute of Genetics and Animal Biotechnology of the Polish Academy of Sciences (Jastrzebiec, Poland). Animals were housed in transparent polycarbonate cages (265 × 207 × 140 mm) in groups of two to five individuals of the same sex. Environmental conditions were kept constant, with a temperature of 20 to 22 °C, relative humidity maintained at 40 to 60%, and a 12:12 h light/dark cycle. Standard rodent chow (caloric value: 13 MJ/kg) and water were provided ad libitum. All procedures were carried out in accordance with national and European regulations for the use of animals in scientific research (approval nr. WAW2/62/2015 [25 June 2015] and WAW2/052/2024 [4 December 2024], II Warsaw Local Ethical Committee).

### 2.3. Sample Collection

To obtain pregnancies, 3–5-month-old females were mated overnight with strain-matched stud males. The day of vaginal plug detection was designated as 0.5 dpc. Pregnant females were euthanized by cervical dislocation at the assigned developmental stage. The uterus was excised and transferred into ice-cold phosphate-buffered saline (PBS) (Roth, Cat#1112.2, Karlsruhe, Germany), and fetuses were immediately dissected under a stereomicroscope. Developmentally retarded and malformed fetuses were excluded at collection; no fetuses were excluded after enrolment. At 17.5 dpc, fetuses were sacrificed immediately, whereas earlier-stage fetuses were processed as a whole. Fetuses were analyzed irrespective of sex. Fetuses were then weighed and allocated to downstream applications: (i) cryopreservation for cresyl violet and immunohistochemistry; or (ii) whole-mount cresyl violet staining for somite counts.

### 2.4. Analysis of Fetal Staging

Fetuses were weighed immediately after collection at 12.5, 15.5, and 17.5 dpc. Developmental staging was assessed with reference to published mouse embryo atlases [24,25]. For staging assessment, whole embryos at 12.5 dpc were fixed in 4% paraformaldehyde (PFA, Sigma-Aldrich, Cat#47608, Burlington, MA, USA) to preserve tissue morphology, subsequently stained with 1% cresyl violet (Sigma-Aldrich Cat#C5042-10G) for 2 h, and finally rinsed in PBS to remove excess dye and reduce background. Whole-mount preparations were then examined and imaged under a stereomicroscope (Nikon, Tokyo, Japan).

### 2.5. Histological Processing

#### 2.5.1. Paraffin Embedding

Fetuses were fixed in 4% PFA at 4 °C for 24 h. Samples were then dehydrated through a graded ethanol series, cleared in xylene (Warchem, Cat#41692, Warszawa, Poland), and infiltrated with molten paraffin (Sigma-Aldrich, Cat#327212) at 62 °C. Tissues were embedded in paraffin blocks, and sections were cut at 12 µm thickness using a rotary microtome (Zeiss Hyrax M25, Oberkochen, Germany).

#### 2.5.2. Cryosectioning

Fetuses were fixed in 4% PFA and cryoprotected in 15% and then 30% sucrose (Sigma-Aldrich, Cat#5973) at 4 °C. Samples were considered as equilibrated when they soaked completely in the sucrose; after that, they were frozen at −80 °C. Samples were then equilibrated for 30 min at −20 °C before being embedded in Cryomatrix (Thermo Fisher Scientific, Cat#6769006, Waltham, MA, USA) and processed at −18 °C into 30 µm thick sections using a cryostat (Leica Jung CM1800, Nussloch, Germany). Sections were collected into multi-well plates containing Tris-buffered saline (TBS) with 0.5% sodium azide (Sigma-Aldrich, Cat#S-2002). All sections were mounted on gelatin-coated SuperFrost glass slides (Bionovo, Cat#8037/1, Emeryville, CA, USA) and stored until further staining procedures.

### 2.6. Histological Staining

#### 2.6.1. Hematoxylin and Eosin (H&E) Staining

Paraffin-embedded sections were deparaffinized in xylene for 15 min and rehydrated through a graded ethanol series (99.8% POCH Cat#396480111, 96% POCH Cat#396420113, and 70%; 5 min each), followed by rinsing in distilled water for 5 min. Nuclear staining was performed using Harris hematoxylin solution (Sigma-Aldrich, Cat#HHS32) for 7 min, after which sections were rinsed in distilled water. Cytoplasmic counterstaining was carried out using 1% Eosin Y alcoholic solution (Sigma-Aldrich, Cat#1.17081) for 30 s, followed by a brief rinse in distilled water. Sections were then dehydrated through ascending ethanol concentrations (70%, 96%, and 99.8%; 1 min each), cleared in xylene for 3 min, and mounted using Distyrene Plasticizer Xylene (DPX; Sigma-Aldrich, Cat#06522). Slides were allowed to dry overnight prior to imaging.

#### 2.6.2. Cresyl Violet (CV/Nissl) Staining

Cresyl violet (Nissl) staining was performed to visualize overall brain morphology and cytoarchitecture. Tissue cryosections were rehydrated through graded ethanol solutions (96% and 70%; 5 min each), followed by rinsing in distilled water for 5 min. Sections were then stained with 0.1% cresyl violet acetate solution (Sigma-Aldrich Cat#C5042-10G) for 9 min. Excess stain was removed by a brief rinse in distilled water (3 min), and differentiation was achieved by immersion in 70% ethanol for 5–8 min. Sections were subsequently dehydrated through ascending ethanol concentrations (96% for 5 min and 99.8% for 3 min), cleared in xylene for 3 min, and coverslipped using DPX mounting medium. Slides were allowed to dry overnight before imaging.

#### 2.6.3. Oil Red O Staining

Cryosections were first incubated in 60% isopropanol (Thermo Fisher Scientific, Cat#149320010) for 3 min to prepare the tissue for lipid staining. Oil Red O is a lipophilic dye that stains neutral lipids and has been used to visualize lipid-rich structures in the nervous system, including myelinated axonal tracts due to their high lipid content [26]. Sections were then immersed in Oil Red O solution (Sigma-Aldrich, Cat#O0625) prepared in 60% isopropanol and incubated for 1 h to allow selective labelling of neutral lipids. After staining, sections were thoroughly rinsed to remove excess dye and reduce background. Finally, coronal sections (bregma −2.00 mm) were mounted using an aqueous mounting medium (Aquamount improved Gurr, BDH, London, UK, Cat#362262H) to preserve lipid staining. For this analysis, three adult brains per strain were processed (male mice, 4 months old).

#### 2.6.4. Immunohistochemistry

Coronal cryosections were incubated with Triton X-100 (Sigma Cat# T9284) 0.5% solution for 30 min, then for 1 h with incubating buffer: 5% donkey serum (Sigma-Aldrich Cat# D9663), 0.3% Triton X-100, bovine serum albumin (Sigma-Aldrich Cat#A7030), and TBS (Sigma, Cat#T5030). Next, the cryosections were incubated overnight with a rabbit anti-EAAT1 (excitatory amino acid transporter 1, also known as GLAST) antibody (Abcam Cat#ab181036, Cambridge, UK) at a concentration of 1:200 in a wet chamber at 4 °C. The next day, the sections were washed with TBS and incubated with a secondary antibody Alexa Fluor 568 (anti-rabbit, Thermo Fisher Scientific, Cat#A-11011) for 1 h at room temperature. After incubation, the sections were washed with TBS and mounted with an anti-fade mounting medium containing 4′,6-diamidino-2-phenylindole (DAPI, ThermoFisher, Cat#00-4959-52). For the immunohistochemical analysis, three brains per strain were processed at each developmental stage (15.5 and 17.5 dpc).

### 2.7. Imaging and Quantitative Measurements

Images of H&E and CV staining were acquired using a Nikon Eclipse E200 microscope (Nikon, Tokyo, Japan) equipped with an Opta-Tech camera (model MI20, Warsaw, Poland) and Capture 2.3 software. Quantitative measurements were performed using ImageJ version 1.54p (NIH). Cortical thickness was assessed at 12.5 and 15.5 dpc in sections containing the choroid plexus. At 15.5 dpc, cortical compartments (VZ, IZ, and CP) were defined based on nuclear density, following the compartment scheme of Aggarwal et al. [23], and measured as both absolute thickness (µm) and as a proportion of total cortical thickness in sagittal sections. IHF length was measured as the distance along the midline between hemispheric walls in coronal sections, matched between strains and stages at comparable rostro-caudal levels. For cortical layering, nine fetuses per strain per developmental stage were analyzed (three fetuses from each of three independent litters). Interhemispheric fissure length (cresyl-violet sections) was measured in all available fetuses from three litters per strain per stage: *n* = 9 (B6) and 9 (BTBR) at 15.5 dpc, and 15 (B6) and 13 (BTBR) at 17.5 dpc. The number of sections analyzed per fetus was 2–3 for interhemispheric fissure length, *n* = 2–3 for total cortical thickness and compartment proportions, and *n* = 3 for the GLAST immunohistochemical analysis. Multiple measurements obtained from sections within the same fetus were averaged so that each fetus contributed a single value to statistical analyses. Confocal imaging was performed to acquire high-resolution fluorescence images of immunostained coronal sections. Images were collected using a Nikon confocal microscope (Nikon A1R) equipped with 405-, 488-, 568-, and 647 nm lasers. All samples within an experiment were imaged using standardized acquisition settings. Confocal image analysis was performed using Imaris 9.8.2 software. Standardized processing parameters were applied across all confocal scans. Investigators were not blinded to strain during image acquisition and quantification, as BTBR and C57BL/6J brains are morphologically distinguishable on sight.

### 2.8. Statistical Analyses

All statistical analyses were performed using GraphPad Prism 11.0.2/92. At least three independent litters per strain at each developmental stage were considered. Three fetuses per litter were randomly selected from 3 to 5 litters (*n* = 9–15 fetuses per strain per stage), with *n* varying across different analyses. The individual fetus was the experimental unit. Where several sections were measured within a fetus, values were averaged so that each fetus contributed a single value. A nested (hierarchical) model with litter as a random factor was first fitted and confirmed that the between-litter variance component did not differ from zero (likelihood ratio test, *p* > 0.99); fetuses were therefore treated as independent replicates, and all comparisons were performed at the fetus level. Total cortical thickness (12.5 and 15.5 dpc) and each cortical compartment proportion (15.5 dpc) were compared between strains by per-compartment nested (hierarchical) *t*-tests with litter as a random factor (*n* = 9 fetuses per strain). The estimated between-litter variance component was zero for the ventricular zone and non-zero for the intermediate zone and cortical plate. Interhemispheric fissure length was analyzed by two-way ANOVA (factors: strain and developmental stage; fetus as the experimental unit), followed by Tukey’s multiple-comparison test. Fetal weight was compared between strains at each developmental stage and somite number at 12.5 dpc by Welch’s *t*-test. Data are presented as mean ± SEM. Normality was assessed using the Shapiro–Wilk test, and homogeneity of variance was evaluated using Levene’s test. Statistical significance was defined as α = 0.05. Reporting adheres to the ARRIVE 2.0 guidelines (see Supplementary Materials).

## 3. Results

### 3.1. Confirmation of Complete Callosal Agenesis in Adult BTBR Mice

**Figure 1:**
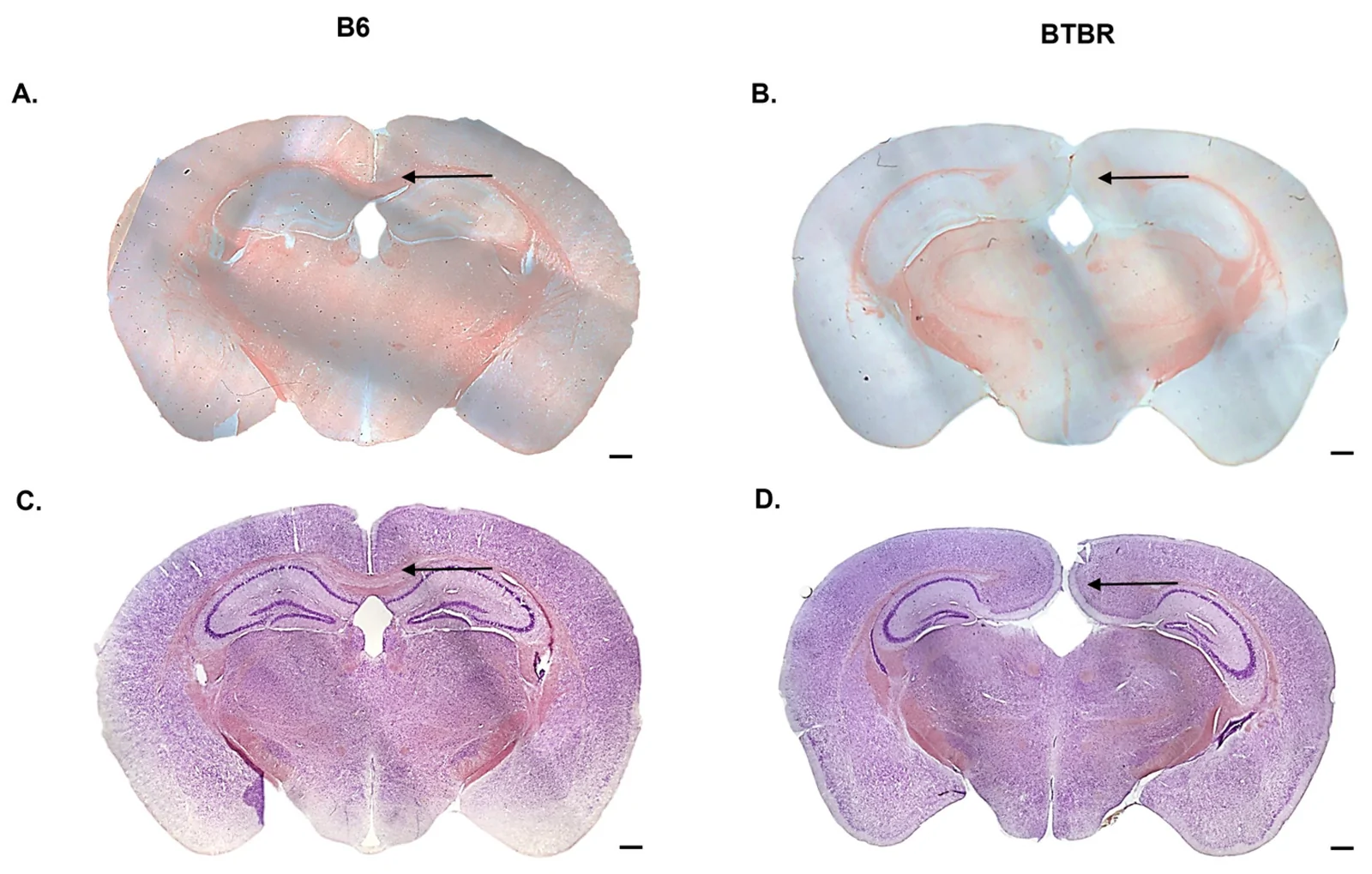
Comparative analysis of adult BTBR and B6 brains. Oil Red O staining of B6 (**A**) and BTBR (**B**) brains, and Oil Red O with cresyl violet counterstain of B6 (**C**) and BTBR (**D**) brains, illustrating complete corpus callosum agenesis in BTBR mice (arrows). *n* = 3 animals per group. Figures obtained by stitching multiple microphotographs. (Scale bar: 500 μm).

As expected, all adult BTBR mice examined in this study exhibited complete agenesis of the corpus callosum, whereas B6 mice displayed a normal callosal structure (Figure 1). This observation confirms that the characteristic BTBR phenotype was fully penetrant in our colony and provides an anatomical reference for the subsequent prenatal analyses.

### 3.2. BTBR and B6 Fetuses Have Similar Somite Count but Different Body Weight

**Figure 2:**
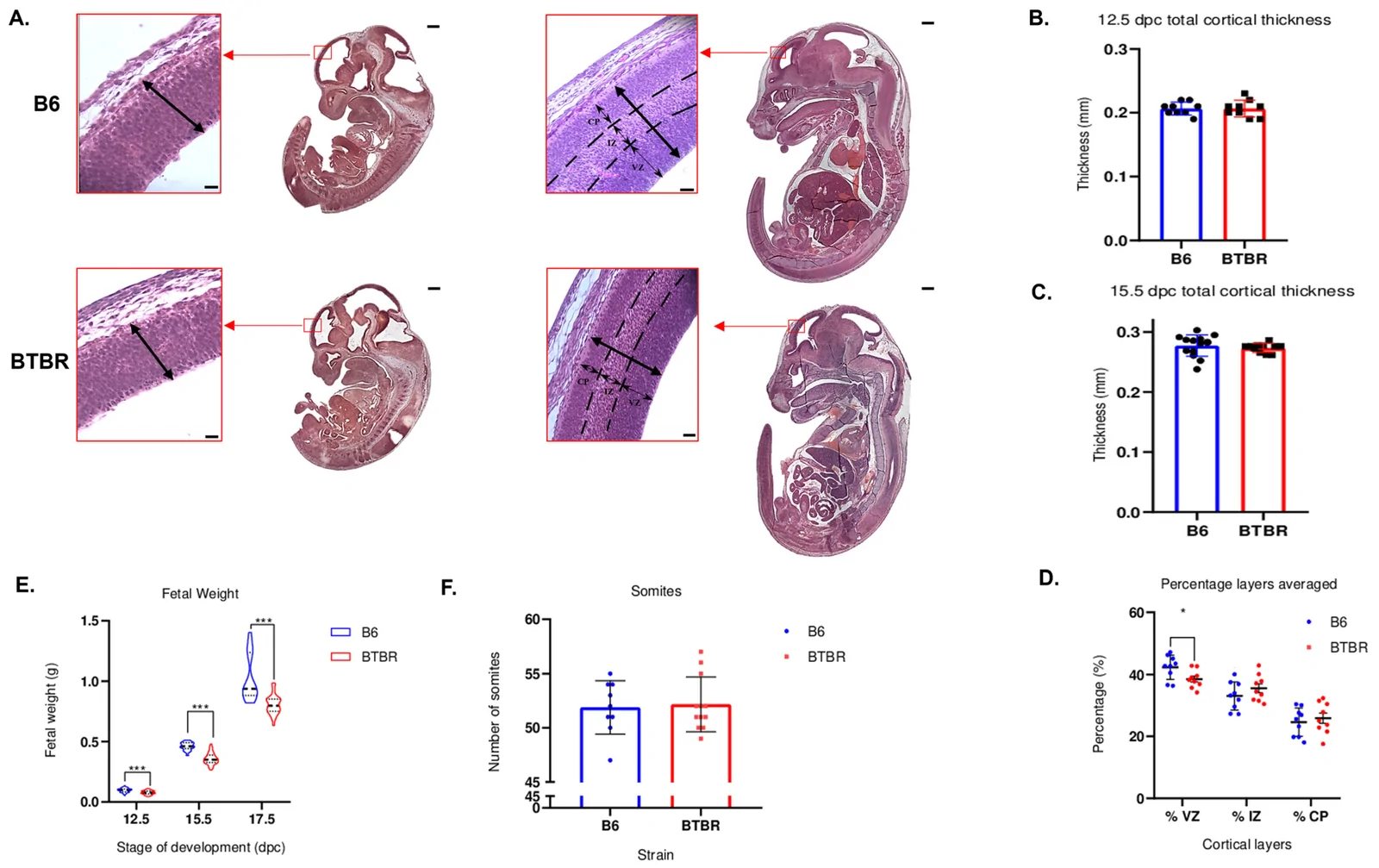
Cortical thickness across developmental stages. (**A**) Histological images of developing cortex in B6 and BTBR strains at 12.5 and 15.5 dpc. Double-headed arrows represent the measured thickness. (**B**) Total cortical thickness (mm) at 12.5 dpc in C57BL/6J (B6) and BTBR T+ Itpr3tf/J (BTBR) strains, analyzed using a nested *t*-test. (**C**) Total cortical thickness (mm) at 15.5 dpc in B6 and BTBR strains, analyzed using a nested *t*-test. (**D**) Proportional thickness of the ventricular (VZ), intermediate (IZ) and cortical-plate (CP) zones at 15.5 dpc, expressed as % of total cortical thickness (mean ± SEM). *n* = 9 fetuses per strain (3 litters × 3 fetuses per litter); strains compared by nested *t* test with litter as the random (nesting) factor. Residual degrees of freedom differ among compartments (VZ, df = 16; IZ and CP, df = 4) because the estimated between-litter variance component differed by compartment. VZ, *p* = 0.032 (*); IZ, *p* = 0.43; and CP *p* = 0.63. (Scale bar full fetus: 300 μm; scale bar cortex: 20 μm). (**E**) Fetal weight of B6 and BTBR across the developmental stages. Strains were compared at each stage by Student’s *t*-test: 12.5 dpc, [t(157) = 7.726, *p* < 0.0001]; 15.5 dpc, [t(57.8) = 10.1, *p* < 0.0001]; 17.5 dpc, [t(13.52) = 3.937, *p* = 0.0016], *** indicates *p* < 0.002. (**F**) Comparison of the somite numbers counted in BTBR and B6 fetuses at the 12.5 dpc stage of development (*p* = 0.8036).

To determine whether delayed brain maturation might underlie the BTBR callosal phenotype, we compared general developmental indices between strains from 12.5 to 17.5 dpc. BTBR fetuses were significantly lighter than B6 fetuses at equivalent dpc (Figure 2E; 12.5 dpc, [t(157) = 7.726, *p* < 0.0001]; 15.5 dpc, [t(57.8) = 10.1, *p* < 0.0001]; 17.5 dpc, [t(13.52) = 3.937, *p* = 0.0016]). However, developmental staging by somite counts revealed no significant differences (Figure 2F). At 12.5 dpc, BTBR embryos had somite numbers strictly comparable to those of B6 controls.

### 3.3. Altered Layer Compartmentalization in the Developing Forebrain Neocortex

Next, we examined the lateral cortical laminar development at mid-gestation at 12.5 and 15.5 dpc. The total radial thickness of the neocortex was statistically similar between BTBR (0.202 mm ± 0.01) and B6 (0.206 mm ± 0.01) fetuses, indicating preserved overall cortical growth [nested *t*-test; t(16) = 0.22; *p* = 0.83] (Figure 2A,B). At 15.5 dpc, overall cortical thickness did not differ between strains (t(16) = 0.66, *p* = 0.51) (Figure 2C), but the proportional allocation of tissue among cortical compartments did (Figure 2D). The ventricular zone occupied a significantly smaller fraction of total cortical thickness in BTBR than in B6 fetuses (38.5% vs. 42.3%; −3.7 percentage points, 95% confidence interval (CI) −7.1 to −0.4; nested *t*-test, t(16) = 2.35, *p* = 0.032). This reduction was accompanied by reciprocal but non-significant increases in the intermediate zone (35.6% vs. 33.1%; t(4) = 0.88, *p* = 0.43) and cortical plate (25.9% vs. 24.6%; t(4) = 0.51, *p* = 0.63), indicating a redistribution among compartments rather than a change in overall cortical size.

### 3.4. Persistent Interhemispheric Fissure Retention Precedes Commissural Failure

**Figure 3:**
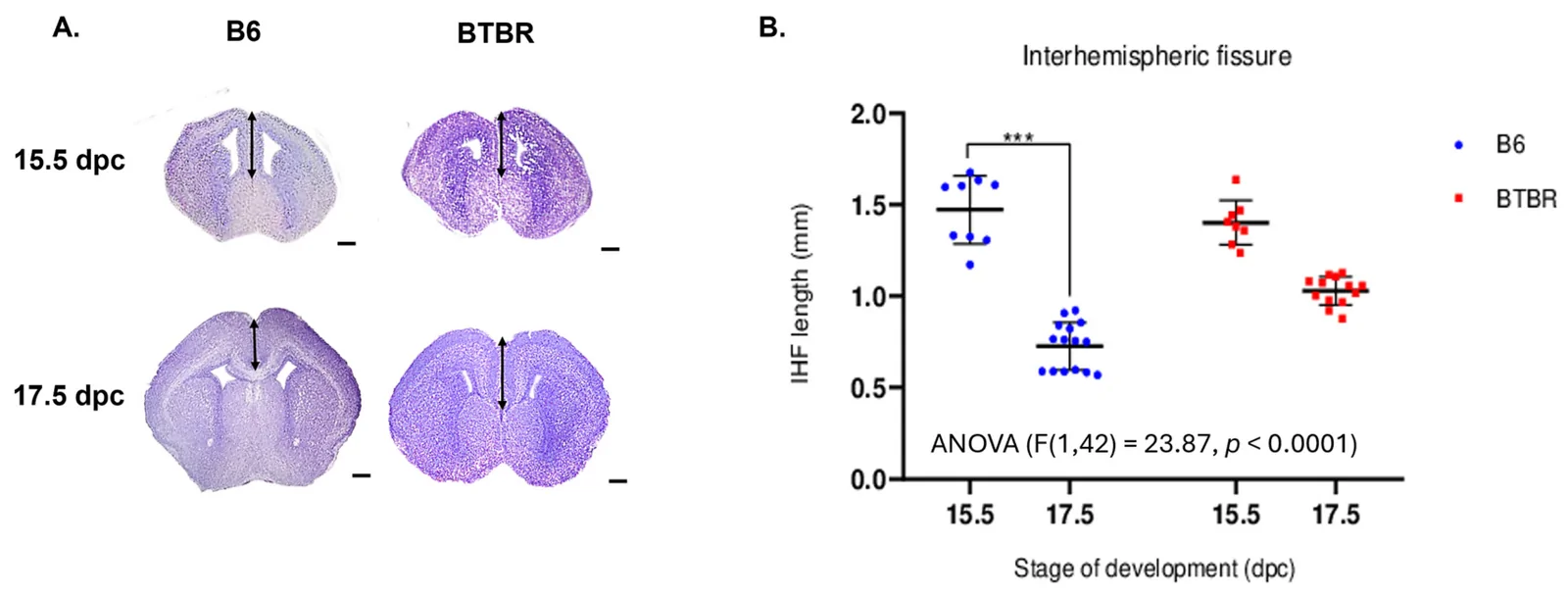
Comparison of the interhemispheric fissure (IHF) at 15.5 and 17.5 dpc between BTBR and B6 fetuses. (**A**) Cresyl violet staining of coronal brain sections showing the interhemispheric region in B6 and BTBR fetuses at 15.5 and 17.5 dpc. (**B**) Quantification of interhemispheric fissure (IHF) length across developmental stages in B6 and BTBR strains. Data are expressed as mean ± SEM. Statistical analysis was performed using two-way ANOVA with Tukey’s multiple-comparison test. Asterisks indicate significant differences between strains and developmental stages (*** *p* < 0.001). *n* = 9 (B6) and 9 (BTBR) fetuses at 15.5 dpc, and 15 (B6) and 13 (BTBR) fetuses at 17.5 dpc; 3 litters per strain per stage. (Scale bar: 250 μm).

The persistence of the IHF in BTBR mice has previously been reported and has been linked to defective midline remodeling [21]. In normal B6 development, the IHF remodels and shortens at specific developmental stages: 15.5 and 17.5 dpc (Figure 3A). BTBR fetuses, in contrast, failed to shorten the fissure to the same extent. Two-way ANOVA (strain × developmental stage; fetus as the experimental unit) revealed a significant strain × stage interaction (F(1,42) = 23.87, *p* < 0.0001) and a strong main effect of developmental stage (F(1,42) = 207.1, *p* < 0.0001); the main effect of strain was smaller (F(1,42) = 8.599, *p* = 0.0054), reflecting the near-identical IHF length of the two strains at 15.5 dpc (Figure 3B). Tukey’s multiple-comparison test confirmed that B6 and BTBR did not differ at 15.5 dpc (*p* = 0.597), whereas at 17.5 dpc the BTBR fissure remained significantly longer than in B6 (mean difference 0.303 mm; 95% CI 0.173–0.433; *p* < 0.0001). Both strains shortened the IHF between 15.5 and 17.5 dpc, but shortening was less complete in BTBR (*p* < 0.0001).

### 3.5. Spatial Disorganization of GLAST+ Midline Cell Populations

**Figure 4:**
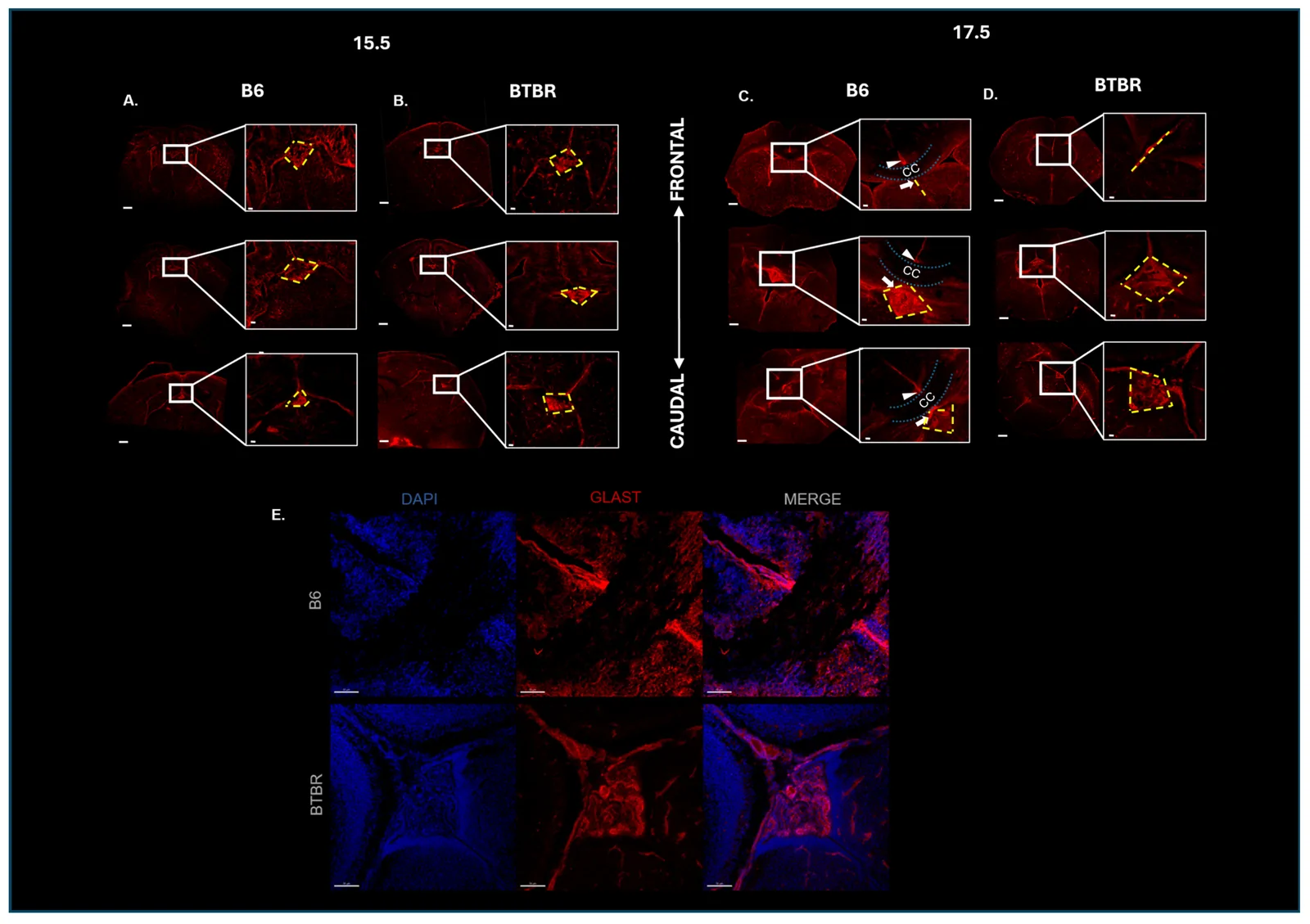
Immunohistochemical detection of GLAST (EAAT1) in fetal brains of B6 and BTBR mice at 15.5 and 17.5 dpc, illustrating GLAST+ immunoreactive cell populations at the midline. Each panel shows a rostrocaudal series with a high-magnification inset. Panel (**A**) shows B6 at 15.5 dpc; Panel (**B**), BTBR at 15.5 dpc; Panel (**C**), B6 at 17.5 dpc; and Panel (**D**), BTBR at 17.5 dpc. CC, corpus callosum, highlighted with dotted lines; arrowheads indicate the indusium griseum; arrows indicate the midline zipper glia (MZG). Dashed lines indicate GLAST+ midline cell populations. Panel (**E**): High-magnification confocal scans of coronal sections 17.5 dpc B6 and BTBR fetal brains stained for GLAST and counterstained with DAPI. (Scale bars panels (**A**–**D**): lower magnification images 200 μm, higher magnification images 100 μm, panel (**E**): 50 μm).

Previous studies have identified abnormalities in midline glial populations and impaired interhemispheric fissure remodeling in BTBR mice [21]. To characterize the organization of GLAST-immunoreactive (GLAST+) midline cells, we examined their spatiotemporal distribution across developmental windows 15.5 and 17.5 dpc. At 15.5 dpc, both B6 and BTBR brains displayed comparable primordial GLAST+ cells assemblies at the cortical midline, suggesting that the initial formation of the GLAST+ midline primordium is conserved in both strains (Figure 4A,B). By 17.5 dpc, however, their organizational trajectories radically diverged. In B6 controls, GLAST-positive populations separated into the indusium griseum (IG) and the midline zipper glia (MZG), forming a well-defined cellular scaffold caudal and ventral to the CC, respectively, at 17.5 dpc (Figure 4C). Conversely, BTBR GLAST+ midline structures do not undergo separation into MZG and IG and maintain a clustered GLAST+ population at the midline. Consequently, the GLAST-positive IG was not properly established, and the MZG populations appeared grossly disorganized and ventrally displaced (Figure 4D,E). These observations indicate that the midline phenotype in BTBR mice involves a failure of GLAST+ midline cell reorganization during the critical 15.5 to 17.5 dpc window and are consistent with a contribution of this midline phenotype to the persistence of the IHF in the BTBR fetus.

## 4. Discussion

This study examined whether structural abnormalities associated with corpus callosum agenesis in BTBR mice can be detected prenatally. We found that overall cortical thickness and embryonic staging were comparable between strains, but BTBR fetuses exhibited altered laminar thickness of the VZ, persistent retention of the IHF, and abnormal organization of GLAST-positive midline structures. These observations indicate that abnormalities in midline morphogenesis are already established during fetal development and occur in parallel with more subtle changes in cortical organization. Our observations indicate that while generalized somatic growth proceeds similarly in BTBR and B6 controls, as measured by somite number at 12.5 dpc, BTBR fetuses exhibit subtle proportional shifts in laminar cortical compartmentalization alongside pronounced spatial disorganization of GLAST+ midline cell populations. Somite counts rule out global developmental delay or acceleration as the cause of neuroanatomical abnormalities, despite smaller body weight in BTBR fetuses. Whether the reduced fetal weight of BTBR fetuses is mechanistically related to the callosal phenotype cannot be resolved by the present data. Previous studies report a correlation between fetal growth restriction and birth weight with CC size reduction in human populations, supported by animal studies on uterine artery ligation as a model of fetal growth restriction [27]. BTBR conceptuses show growth restriction alongside altered placental architecture [28] and altered metabolism and epigenetic state [29], which may affect both fetal growth and neurodevelopment. However, strong evidence indicates that the etiological and neuropathogenic causes of CC agenesis in this strain are genetic, including the mutation in the *DRAXIN* gene [21].

Overall cortical thickness at mid-gestation was comparable between strains. However, detailed laminar analysis at 15.5 dpc revealed a relatively thinner VZ in BTBR fetuses. Importantly, these alterations were observed despite preserved total cortical thickness, suggesting that developmental differences in BTBR are not driven by generalized growth deficits but rather by changes in the relative allocation of cells within cortical compartments. Although these findings do not directly explain CC agenesis, they identify previously underreported prenatal alterations in cortical organization that may influence the production and maturation of callosal projection neurons. Future studies incorporating cell-type-specific markers, proliferation assays, and lineage tracing approaches will be necessary to determine the cellular mechanisms underlying these compartmental changes. Extending the compartment analysis to 17.5 dpc, when the cortical layers are established, would be informative but would require layer-specific molecular markers rather than nuclear density alone. Such changes likely reflect alterations in the balance of neuronal progenitor proliferation, neuronal differentiation, and/or radial migration [17,30,31,32,33,34,35]. The organization of the VZ, IZ, and CP during mid-gestation reflects the balance of progenitor proliferation and neuronal migration, which are known to be implicated in autism development [36]. These cortical variations align with the polygenic background of the BTBR strain. Downregulation of Ext1 in BTBR mice reduces heparan sulfate levels within the germinal niche, potentially impairing morphogen retention necessary for progenitor maintenance. This provides a plausible explanation for subtle apical progenitor depletion and VZ thinning [37]. In parallel, the naturally occurring Disc1 mutation carried by BTBR mice may influence radial migration kinetics, contributing to laminar defects in the cortex [38]. Importantly, these lateral cortical alterations appear to occur independently of the severe midline fusion defect, indicating concurrent but mechanistically distinct developmental deviations [39].

Our observations on persistent IHF retention and abnormal midline GLAST+ cell organization are largely consistent with previous studies demonstrating defective fissure remodeling and disrupted MZG development in BTBR mice. The present work therefore serves primarily as an independent replication of these findings using our colony and experimental pipeline. Such replication is valuable because it confirms the robustness and reproducibility of the BTBR phenotype across laboratories and experimental settings. The most compelling finding of our study concerns midline GLAST+ structure organization and IHF remodeling. In B6 fetuses, midline glial populations, including the glial wedge, IG, and MZG, form highly organized assemblies that compact the IHF and provide a permissive substrate for pioneer callosal axons [40,41]. These astroglial populations derive from radial glia undergoing differentiation and somal translocation, processes regulated by fibroblast growth factor signaling and transcription factors such as *Nfia/b* that control glial morphology and positioning [22,42]. Midline astroglia further shape commissural development through secretion of repulsive Slit ligands acting via Robo receptors [43] and attractive cues such as Netrin-1 signaling through DCC [21,44,45]. Disruption of these coordinated mechanisms leads to callosal agenesis or severe dysgenesis, as demonstrated in multiple genetic models where glial wedge formation or guidepost positioning is impaired [42,46]. In addition, BTBR fetuses displayed gross disorganization and ventral displacement of GLAST+ cell clusters at the midline, accompanied by persistent retention of the IHF between 15.5 and 17.5 dpc. These findings are consistent with a failure of astroglia-mediated remodeling. Emerging work demonstrates that remodeling of the leptomeninges is a prerequisite for CC formation in mice and humans, although the mechanisms underlying the formation of MZG and IG are still unclear. The disorganized GLAST+ aggregates observed in our study are consistent with the recent identification of an 8-base-pair deletion in the *Draxin* gene in BTBR mice. DRAXIN regulates MZG translocation and suppresses leptomeningeal overproliferation; its loss results in defective intercalation and persistence of leptomeningeal tissue within the fissure [21,45,47,48,49].

Midline astroglial disorganization may disrupt not only physical architecture but also local biochemical signaling. In a normally organized state, astroglial structures establish spatially restricted gradients of guidance cues that direct pioneer axons toward the midline crossing region [21,43,44,45]. Disruption of this scaffold deprives axons of essential directional cues, potentially contributing to their stalling and redirection into longitudinal Probst bundles. In addition, regulatory genes such as *Gli3* and *Tcf4* coordinate neuron and astroglia positioning at the midline; mutations in these pathways result in callosal agenesis and mislocalized guideposts [50,51]. Astroglia also engage in metabolic coupling with developing neurons, including lactate shuttling critical for axonal growth, and broader disturbances in glial function align with reports of metabolic and immune dysregulation in BTBR [52,53,54,55].

In humans, reduced corpus callosum area has been reported in autism cases, although studies differ in its magnitude and regional distribution [3]. Nevertheless, CC dysgenesis is not part of the defined diagnostic criteria for autism [4,5], but it is known to reflect wider connectome alterations, with heterotopic callosal connections having an essential role in determining the global properties of brain networks [56]. It is therefore plausible that structural commissural defects and behavioral abnormalities in BTBR represent parallel but dissociable outcomes of its polygenic landscape. While deletion of *Draxin* likely drives the midline morphogenetic defect, additional genetic loci likely contribute independently to behavioral phenotypes [57].

From a methodological standpoint, fetuses were analyzed irrespective of sex due to the absence of overt external sexual dimorphism during the 12.5 to 17.5 dpc window. Given the male bias in human neurodevelopmental disorders and the possibility of sex-dependent penetrance of polygenic mutations, the absence of sex-specific analysis represents a limitation of the present study. Future research incorporating retrospective genotyping will be required to determine whether cortical progenitor dynamics and/or astroglial disorganization exhibit sexually dimorphic variability. A further limitation is that behavioral phenotyping could not be performed in the animals analyzed here, as all were fetuses collected between 12.5 and 17.5 dpc. The autism-like behavioral phenotype of this colony has been characterized previously [28,58], and future work will be required to relate the prenatal midline phenotype described here to behavioral outcomes. Furthermore, the midline cell populations described here were identified using a single marker (i.e., GLAST), thereby establishing the spatial organization of GLAST-immunoreactive cells at the midline rather than the identity of the cells that comprise them.

While generalized somatic growth and lateral cortical lamination proceed with only subtle strain differences, the morphogenetic sequence governing midline GLAST+ cell reorganization is profoundly disrupted during the critical 15.5 to 17.5 dpc window.

## 5. Conclusions

In conclusion, prenatal brain abnormalities identified in BTBR fetuses encompassed the failure of interhemispheric fissure shortening accompanied by disruption of midline GLAST+ cell structure remodeling at mid–late developmental windows. In contrast, cortical abnormalities were subtle and consisted primarily of altered proportions of the ventricular zone, without changes in overall cortical thickness or somite counts. These findings emphasize the central role of prenatal developmental dynamics in establishing commissural connectivity and offer a structural framework for future mechanistic studies linking prenatal abnormalities with specific neuroanatomical phenotypes characterizing the BTBR mouse.

## Data Availability

The data underlying this article are available in the article and in its online Supplementary Materials, or will be shared upon reasonable request to the corresponding author.

## Supplementary Materials

The following supporting information can be downloaded at https://www.mdpi.com/article/10.3390/biology15161425/s1, the ARRIVE 2.0 checklist.
