# Supplementary material for: Fetal Cortical Layers and Midline Histological Alterations in the BTBR Mouse Model of Neurodevelopmental Disorders
Source: Biology (Basel). 2026 Aug 18;15(16):1425. doi: 10.3390/biology15161425 (PMC13509614; doi:10.3390/biology15161425)
Supplement: Supplementary file 1 [file biology-15-01425-s001.zip › biology-4473501-supplementary.pdf]

NOTE: Please save this file locally before filling in the table, DO NOT work on the file within your internet browser as changes will not be saved. Adobe Acrobat Reader (available free here) is recommended for completion.

The ARRIVE guidelines 2.0: author checklist

The ARRIVE Essential 10

These items are the basic minimum to include in a manuscript. Without this information, readers and reviewers cannot assess the reliability of the findings.

Item		Recommendation	Section/line number, or reason for not reporting
**Study design**	1	For each experiment, provide brief details of study design including:	
		a. The groups being compared, including control groups. If no control group has been used, the rationale should be stated.	strain comparison: BTBR vs C57BL6/J. Stage comparison.
		b. The experimental unit (e.g. a single animal, litter, or cage of animals).	The individual fetus was the experimental unit; litter was included as a random factor in the nested models (Section 2.8).
**Sample size**	2	a. Specify the exact number of experimental units allocated to each group, and the total number in each experiment. Also indicate the total number of animals used.	Three fetuses per litter, drawn from 3-5 litters per strain per developmental stage (n = 9-15 fetuses per strain per stage; n varies by analysis - see item 3c).
		b. Explain how the sample size was decided. Provide details of any *a priori* sample size calculation, if done.	power analysis based on published literature
**Inclusion and exclusion criteria**	3	a. Describe any criteria used for including and excluding animals (or experimental units) during the experiment, and data points during the analysis. Specify if these criteria were established *a priori*. If no criteria were set, state this explicitly.	developmentally retarded and malformed fetuses were excluded from the study
		b. For each experimental group, report any animals, experimental units or data points not included in the analysis and explain why. If there were no exclusions, state so.	no exclusion after fetuses enrolled
		c. For each analysis, report the exact value of *n* in each experimental group.	Cortical thickness and compartment proportions: n = 9 per strain per stage (3 litters x 3 fetuses). Interhemispheric fissure length: n = 9 (B6) and 9 (BTBR) at 15.5 dpc; n = 15 (B6) and 13 (BTBR) at 17.5 dpc [illegible]
**Randomisation**	4	a. State whether randomisation was used to allocate experimental units to control and treatment groups. If done, provide the method used to generate the randomisation sequence.	fetuses were randomly selected from each litter
		b. Describe the strategy used to minimise potential confounders such as the order of treatments and measurements, or animal/cage location. If confounders were not controlled, state this explicitly.	animals were all housed under identical conditions.
**Blinding**	5	Describe who was aware of the group allocation at the different stages of the experiment (during the allocation, the conduct of the experiment, the outcome assessment, and the data analysis).	All operators were aware of group allocation. Blinding was not feasible because BTBR and C57BL/6J brains are morphologically distinguishable on inspection (Section 2.7).
**Outcome measures**	6	a. Clearly define all outcome measures assessed (e.g. cell death, molecular markers, or behavioural changes).	(i) fetal body weight at 12.5, 15.5, and 17.5 dpc and somite counts at 12.5 dpc; (ii) total cortical thickness at 12.5 and 15.5 dpc; (iii) relative thickness of the cortical compartments: ventricular zone (VZ) [illegible]
		b. For hypothesis-testing studies, specify the primary outcome measure, i.e. the outcome measure that was used to determine the sample size.	corpus callosum agenesis (100% penetrance in BTBR mice)
**Statistical methods**	7	a. Provide details of the statistical methods used for each analysis, including software used.	All statistical analyses were performed using GraphPad Prism ver.11.0.2/92. At least three independent litters per strain at each developmental stage were considered. Three fetuses were randomly selected from [illegible]
		b. Describe any methods used to assess whether the data met the assumptions of the statistical approach, and what was done if the assumptions were not met.	Normality was assessed using the Shapiro–Wilk test, and homogeneity of variance was evaluated using Levene's test.
**Experimental animals**	8	a. Provide species-appropriate details of the animals used, including species, strain and substrain, sex, age or developmental stage, and, if relevant, weight.	Mus musculus<br>Strains: (i) BTBR T+ Itpr3tf/J, and C57BL/6J Adult brain were obtained from male mice aged 4 months. Fetuses were studied irrespective of their sex.
		b. Provide further relevant information on the provenance of animals, health/immune status, genetic modification status, genotype, and any previous procedures.	Animals derive from the colony of the Institute of Genetics and Animal Biotechnology of the Polish Academy of Science, Jastrzębiec, Poland. Maintained under inbreeding protocols. No previous [illegible]
**Experimental procedures**	9	For each experimental group, including controls, describe the procedures in enough detail to allow others to replicate them, including:	no procedure were conducted on live animals. Pregnant mice were humanely sacrificed by cervical dislocation.
		a. What was done, how it was done and what was used.	N.A.
		b. When and how often.	N.A
		c. Where (including detail of any acclimatisation periods).	N.A
		d. Why (provide rationale for procedures).	
**Results**	10	For each experiment conducted, including independent replications, report:	within the manuscript
		a. Summary/descriptive statistics for each experimental group, with a measure of variability where applicable (e.g. mean and SD, or median and range).	
		b. If applicable, the effect size with a confidence interval.	within the manuscript
